# Properties of Pectin Extracted from Vietnamese Mango Peels

**DOI:** 10.3390/foods8120629

**Published:** 2019-12-01

**Authors:** Hoa H. D. Nguyen, Ha V. H. Nguyen, Geoffrey P. Savage

**Affiliations:** 1 Food Technology Department, Biotechnology School, International University, Vietnam National University, Ho Chi Minh City 700000, Vietnam; hoanghoa19sep@gmail.com; 2 Food Group, Department of Wine, Food and Molecular Biosciences, Agriculture and Life Sciences, Lincoln University, Canterbury 7674, New Zealand; Geoffrey.Savage@lincoln.ac.nz

**Keywords:** mango peel pectin, mango cultivars, maturity stages, Fourier-transform infrared spectroscopy (FTIR), rheological property

## Abstract

This study was carried out to investigate the properties of pectin extracted from Vietnamese mango peels that have been discarded as waste. Three different mango cultivars named Hoa Loc, Ghep and Cat Chu at three different maturities were studied. Pectin extracted from immature, ripe and overripe stages ranged from 18.4 to 31.7 g/100 g dry weight (DW); the highest yields were obtained from the ripe fruits. Ghep peels contained the highest pectin amounts which ranged from 24.2 to 31.7 g/100 g DW, followed by Cat Chu (19.2 to 26.5 g/100 g DW) and Hoa Loc peels (18.4 to 24.1 g/100 g DW). Except for degree of esterification and emulsion capacity, other properties of the extracted pectin including water holding capacity, solubility and emulsion stability were significantly affected by the fruit maturation. Varieties, solubility, degree of esterification, emulsion activity and emulsion stability of the pectin isolated from the three cultivars ranged from 77.4% to 86.0%; 50.3% to 55.8%; 11.8% to 34.3% and 28.5% to 94.5%, respectively. Fourier-transform infrared results showed that almost all collected pectin samples could be classified as the high methoxyl pectin. Rheology data indicated viscosity of the isolated pectin was strongly dependent on temperatures.

## 1. Introduction

Pectins are complex colloidal acid polysaccharides [1]. Pectic substances have high molecular weights and can be classified into four groups: protopectin, pectic acid, pectinic acid and pectins [2]. Pectins are widely used as thickening and stabilizing agents in beverages, dairy products and confections due to their gelation properties [1,2]. They are also applied in pharmaceutical products to reduce the absorption of cholesterol [3], control haemorrhage and effect of poisoning of toxic cations [1]. In the upper intestine, pectin can reduce postprandial satiety, the absorption of nutrients and the motility of the intestinal tract [4].

High levels of pectins are found in almost all fruit fractions including peels, pulp and kernels [5,6,7]; however, the pectin yield depends very much on the cultivars and the maturity stages when a fruit is harvested and analysed [8,9].

The major constituent of pectin molecules is poly (1-4)-α-D-galacturonan, which contains carboxyl groups presenting in either free acid or methyl ester forms. The result of dividing esterified carboxylic acid units into total carboxylic acid groups in the pectin chain has been defined as the degree of esterification (DE). The DE value of pectin is an important functional property that significantly influences its commercial use as a gelling or thickening agent. The degree of esterification is strongly influenced by calcium cations, sugar and acid concentrations. Depending on the proportion of the esterified groups, pectin is classified into low methoxyl pectin (LMP) (DE < 50%) and high methoxyl pectin (HMP) (DE > 50%) [1]. Structural differences between pectin molecules affect the gelling mechanisms of LMP and HMP; hence, their applications in foodstuffs are also different.

Although pectin has been commonly extracted from agricultural byproducts, the number of sources used for extracting commercial pectin production is limited [10]. The gel forming ability of pectin strongly depends on the molecular size and degree of esterification of each different byproduct; therefore, the isolation and characterization of pectin from each new source are important for the development of a new pectin product.

In Vietnam, mangoes (*Mangifera indica* L.) are grown in most southern regions (approximately 75,000 hectares in total) producing 0.5 million tons of mangoes/year of which 70% is used for processing [11]. Mango processing releases large amounts of byproducts consisting of 35–60% of total fruit weight. This fraction includes peels, stones and sometimes parts of perishable pulp [12]. Dorta et al. [13] reported that the world mango production produces approximately 75,000 tons of mango wastes/year. As mango peels constitute 15–20% of the fruit weight, the production of mango waste in Vietnam would be between 50,000 and 70,000 tons/year. Currently, these wastes have been used for animal feeding or dumped as rubbish; therefore, there is an urgent need to use this waste resource effectively. Many experiments have been carried out using mango byproducts in order to reduce the negative effects of disposing this waste product. Interestingly, it has been found that mango peels are a good source of dietary fiber including pectin, polyphenols, carotenoids and other bioactive compounds that have positive influences on human health [13,14]. Extractions of pectin from mango peels have been carried out by Al-Sheraji et al. [5] and Ajila and Prasada Rao [14]; however, effects of biological factors including maturity stages and cultivars on properties of pectin have been overlooked. Moreover, it is noticeable that Vietnamese processors have to pay more than United States Dollar 4 million annually to import pectin from other countries [15]. Therefore, analysing physiochemical properties of pectin extracted from a few common cultivars of mango is an important step to improve the utilisation of this useful byproduct.

## 2. Materials and Methods 

### 2.1. Sample Preparation

In brief, 180 kg of three different cultivars of healthy mangoes; Hoa Loc, Cat Chu and Ghep were harvested from a farm in Tien Giang province, Vietnam in November 2015. The three cultivars harvested at three different maturity stages: pre-mature, mature and ripe were transported to the laboratory of the International University, Thu Duc District, Ho Chi Minh City and processed further on the same day. The fruits were carefully washed under running tap water to remove any dirt, insects and debris on surface of the fruits. Peels then were separated from fruits using a stainless-steel knife and dried in an oven (WiseVen, Wisd Laboratory Instruments, Gangwon-do, Korea) at 60 °C for 24 h. The dried samples were ground into a fine powder using an A11 grinder (IKA, Selangor, Malaysia) and then packed in individual plastic bags and stored in desiccators until analysis commenced.

### 2.2. Pectin Extraction from Dried Mango Peel Powder

Pectin extractions were carried out following the procedure of Nguyen and Savage [6] with some modifications. The dried peel powder of each cultivar at different maturity stages was mixed with the aqueous solution of citric acid (Merck Sharp & Dohme Corp., Kenilworth, NJ, USA) 1.5% in a ratio 1:40 (*w*/*v*). The mixture was stirred continuously for 20 min and then filtered through four layers of cheesecloth in order to separate the supernatant from the insoluble fraction. Pectin was precipitated by the addition of absolute ethanol (98% purity) with a ratio of 1:2 (*w*/*w*) into the supernatant and kept overnight at room temperature. The precipitated pectin was then washed three times with 75%, 85% and 98% (*v*/*v*) ethanol to remove the soluble impurities. The pellet was then freeze-dried (FreeZone 2.5 L Benchtop Freeze Dry System, Labconco, Kansas city, MO, USA) until a constant weight was obtained.

### 2.3. Pectin Yield

(1) $$\mathrm{The}\;\mathrm{yield}\;\left(\%\right)=\frac{\mathrm{weight}\;\mathrm{of}\;\mathrm{dried}\;\mathrm{pectin}\;\left(g\right)\;\times \;100}{\mathrm{weight}\;\mathrm{of}\;\mathrm{dried}\;\mathrm{peel}\;\mathrm{taken}\;\mathrm{for}\;\mathrm{extraction}\;\left(g\right)}$$

### 2.4. Measurement of the Degree of Esterification

The collected pectin was stored in vacuumed bags at 4 °C before measuring their degree of esterification by Fourier-transform infrared spectroscopy (FTIR) analysis. In detail, FTIR spectra of pectin samples were obtained using a Tensor 27 Spectrophotometer (Bruker AXS GmbH., Karlsruhe, Germany) that measured the absorbance of asymmetrical stretching vibrations of the carboxyl groups (1600–1630 cm^−1^ wavenumber) and carbonyl groups originating from carboxyl and carbomethoxyl groups (1730–1760 cm^−1^ wavenumber) on KBr disks with a 90:10 KBr/pectin ratio. Then, the degree of esterification was calculated using the equation proposed by Singthong et al. [16].

### 2.5. Determination of Water Holding Capacity (WHC) and Solubility of Mango Peel Pectin

These properties were determined using centrifugation techniques by modifying the method of Eastwood et al. [17]. In detail, 0.3–0.5 g of pectin samples were soaked in 20 mL of Nanopure water and left to stand for 1 hour at room temperature before centrifugation at 6000 rpm for 15 min. Then, the supernatant fraction was discarded and the pellets were left to drain during 30 min at ambient temperature. The pellets were dried until obtaining the constant weight.

The changes in weight of pellet were recorded and the WHC and solubility of pectin samples were conducted

(2) $$\mathrm{Water}\;\mathrm{holding}\;\mathrm{capacity}\;=\;\frac{m_{w}-m_{d}}{m_{d}}\;({g\;H}_{2}O/g\;\mathrm{dried}\;\mathrm{pectin})$$

(3) $$\mathrm{Solubility}={\;m}_{i}-\;m_{d}\left(\%\right),$$

where: m_i_: initial weight of dried pectin samples before WHC processing; m_w_: wet weight of pectin samples; m_d_: dried weight of pectin samples after WHC processing.

### 2.6. Determination of the Emulsion Activity and Emulsion Stability

To measure the emulsion activity of the collected pectin, two volumes of prepared pectin gel (60 mL) (0.5% *w*/*v* pectin solution) were thoroughly mixed with the soybean oil (6 mL) [18]. The mixture was then homogenized (HG-15A, Witeg Labortechnik GmbH, Wertheim, Germany) for 1 min and then centrifuged (Z326K, Hermle Labortechnik GmbH., Wehingen, Germany) at 800 g for 10 min. The emulsion activity was calculated as the ratio of the volume of emulsified layer with the volume of whole layer in centrifuge tube. For the determination of the emulsion stability, emulsions prepared by the above procedures were heated at 80 °C for 30 min and cooled to room temperature [18]. Then, the content was centrifuged at 800 g for 10 min. The emulsion stability was measured as the ratio between the remaining emulsified layer in the supernatant and the initial emulsified layer.

### 2.7. Rheological Analysis

Rheological analysis of the pectin solutions was performed in a HAAKE viscometer and rheometer RheoStress 6000 (Thermo Scientific, Waltham, MA, USA). Thermo Scientific HAAKE RheoWin software (Thermo Scientific, USA) was run to record shear stress (τ) and shear rate (γ) values. Pectin (10 g L^−1^) were dissolved in 0.1 mol L^−1^ sodium chlorine solutions (Merck Sharp & Dohme Corp., Kenilworth, NJ, USA) and left for 12 h at room temperature prior to measuring [19]. The flow curves were obtained at different processing temperatures 4 °C, 30 °C and 50 °C. The shear rates from 10.0 to 300 s^−1^ were chosen.

### 2.8. Determination of Viscosity-average Molecular Mass

Dried pectin 0.25; 0.5; 1.0; 1.5 and 2.0 g was dissolved in 100 mL of 0.1 M potassium phosphate (pH: 7.0) [20]. The viscosity measurement was performed at 25 °C using a Cannon-Fenske viscometer tube, size 100 (sigma-aldrich, Germany) with constant k = 0.015. Specific, reduced and intrinsic viscosities were calculated by the following equations:

(4) $$\mathrm{Specific}\;\mathrm{viscosity}:\;\eta_{sp}=\;\frac{t\;-\;t_{0}}{t}$$

(5) $$\mathrm{Reduced}\;\mathrm{viscosity}:\;\eta_{red}=\;\frac{\eta_{sp}}{C}$$

(6) $$\mathrm{Intrinsic}\;\mathrm{viscosity}:\;\eta_{i}=\;\underset{C\to 0}{\lim}\frac{\eta_{sp}}{C},$$

where: *t* is time taken by the solution to flow in viscometer (s); *t_0_* is time taken by the solvent to flow in viscometer; C the concentration of pectin solution (g/ 100 mL) 

The relationship between intrinsic viscosity and molecular mass is described by the Mark–Houwink–Sakurada equation [21]:

(7) $$\eta_{i}=K\times \;M_{w}^{\alpha }$$

where: K and α are constants. At 25 °C, K and α are 1.4 × 10^−6^ and 1.34, respectively [22].

### 2.9. Statistical Analysis

The results were presented as mean of three determinations ± standard error. Two-way analysis of variance (ANOVA) was performed using Minitab software version 16.0 for Windows 7 (Minitab Pty Ltd., Sydney, NSW, Australia) with a level of confidence of 95%.

## 3. Results and Discussion

### 3.1. Pectin Yield

The yield of pectin extracted from the peels of each of the three mango cultivars at ripening stage was significantly higher (*p* < 0.05) when compared to the mature stage (Table 1). The amounts of pectin extracted from the mature stage of the Cat Chu and Hoa Loc cultivars gave intermediate values and significantly lower levels for Ghep. Similar observations were made by Proctor and Peng [23] who noted that amounts of extracted pectin declined steadily before the fruits reached full maturation but increased during fruit ripening. During fruit ripening, polygalacturonase and pectin methyl esterase hydrolyse the pectin backbone and solubilize insoluble protopectin into soluble pectin; consequently, more soluble pectin is produced [24]. However, the activity of pectin methyl esterase was highest in over–ripe and in immature fruit. Meanwhile, the polygalacturonase activity was very low in unripe fruit [24]. Moreover, pectinolytic enzyme activities, especially polygalacturonase, have been reported to be different between fruit cultivars [9]. Therefore, the differences in amounts of pectin observed at different maturity stages and cultivars may be due to the different activities of both pectin methyl esterase and polyglacturonase, which are active during the development of the fruit [8,9,24].

The yield of pectin recovered in this study was much higher than recovered from other mango cultivars, including Améliorée (10.1 g/100 g DW), Mango (15.3 g/100 g DW) [2] and Tommy Atkins (17.6 g/100 g DW) [25].

### 3.2. Water Holding Capacity (WHC)

Water holding capacity, defined as the amount of water held by the fibrous matrix [17], has been shown to be a key physical property of pectin [2]. During ripening, the reduction of insoluble materials of pectic substances is inversely proportional to the increase of water-soluble substances. As a result, the water holding capacity of a fruit would be changed.

The overall mean of water holding capacity of the pectin extracted from the three cultivars of mango was 12.0 ± 0.17 g H_2_O/g DW. The water holding capacity measured at different maturity stages was variable for each of the cultivars but only Ghep showed an increased level when the fruit was fully ripe. It was reported that the side chains of the pectin molecule consist of glucose, arabinose, xylose, galactose, mannose and rhamnose, creating “sugar building blocks” [16]. Furthermore, mangoes are climacteric fruits which have high respiration rates when they are fully mature. At this stage total sugar contents rapidly increase following starch degradation [10]. The high sugar levels coincide with increasing organic acid contents in the mature fruit, causing pectin molecules to no longer repel each other [4]; as a result, they form stable three dimensional networks, allowing more water and sugar molecules to be trapped in the structure of pectin samples extracted [26]. The pectin extracted from this stage is of considerable interest to food processors as it has considerably increased water holding capacity.

However, in the cell wall, organic acids decrease during fruit ripening [10]. This leads to a shortage of protons for balancing the anion carboxylate groups in the pectin structure. Therefore, the approach and interaction among polysaccharide molecules could be suppressed, resulting in an unbalanced network formation which contains weaker bonds [10]. These weak bonding networks may lower the water holding capacity of pectin molecules extracted from fully ripe fruits of Cat Chu and Hoa Loc.

The most interesting feature of the present study is that the WHC in the Vietnamese mango peels was much higher when compared to lemon peels (1.7–1.9 g H_2_O/g DW) [27], yellow passion fruit peels (3.7–4.1 g H_2_O/g DW) [28] and Tommy Atkins mango peels (4.7–6.1 g H_2_O/g DW) [7]. Significant differences in the WHC of pectin extracted from 12 different cultivars of pomegranates [29] and cabbage [3] have also been observed. The WHC of pectin has an important effect of reducing free water in a gel matrix [30] and the pectin samples extracted in this present study show an important potential function as a food ingredient to prevent syneresis of formulated products [30].

### 3.3. Solubility

The solubility of the pectin extracted from the three different cultivars in this study were significantly different (*p* < 0.05): overall the levels increased as the fruits matured. The increase in solubility of the pectin extracted as the fruit matures results from the increase in the pectinolytic enzyme activities, especially polygalacturonase and these have been reported to be different between fruit cultivars [9]. The main effect appears to be variations in the reductions of the pectin chain width and removal of the neutral sugars from the side chains [31]. The increase in solubility of the pectin extracted from the three different mango cultivars as they mature is a very important finding of this study.

At the onset of fruit ripening, the hydrolytic enzymes—polygalacturonase and pectin methyl esterase—break down the pectin chain, producing smaller units [24]. In the early stage of fruit ripening, β–galactosidase enzyme was reported to be responsible for an increasing number of free pectin molecules present in the cell wall [31]. Additionally, β–galactosidase cuts the sugar chains in the pectin structures into shorter pieces, resulting in an increase in the water solubility of the pectin [31]. As ripening proceeds in fruits, the pectin becomes more soluble; this may not be a positive feature in the use of extracted pectin for food processing applications.

### 3.4. Degree of Esterification

Many studies have been carried out to measure the degree of esterification (DE) of pectin extracted from different plant sources [32,33,34], however, investigation of the possible changes in DE in pectin samples extracted from different maturities and cultivars of mangoes using the FTIR method have not been studied.

It was found from the current work that the overall mean DE of the three mango cultivars was 52.6 ± 1.6%; there were significant differences between the cultivars; however, insignificant changes occurred as the cultivars matured (Table 1). These results could be due to differences in the activities of pectin methyl esterase and β–galactosidase. The same phenomenon was also observed between 16 different cultivars of tomatoes [35] and two apple cultivars [36].

The overall DE values of the three cultivars investigated in this study were higher than those of pectin, extracted using the same acid hydrolysis extraction method, from apple pomace (22.2%) [32], dragon fruit peels (31.1–47.0%) [33] and cocoa pod husk (40.3%) [34]. The pectin extracted in this study could be considered to be high–methoxyl pectin, which is more desirable and useful in commercial food production where pectin with mean DE values > 50% is of considerable use in the manufacture of low pH products like fruit jellies or low-methoxyl pectin through de-esterification process [1].

### 3.5. FTIR Analysis

**Figure 1:**
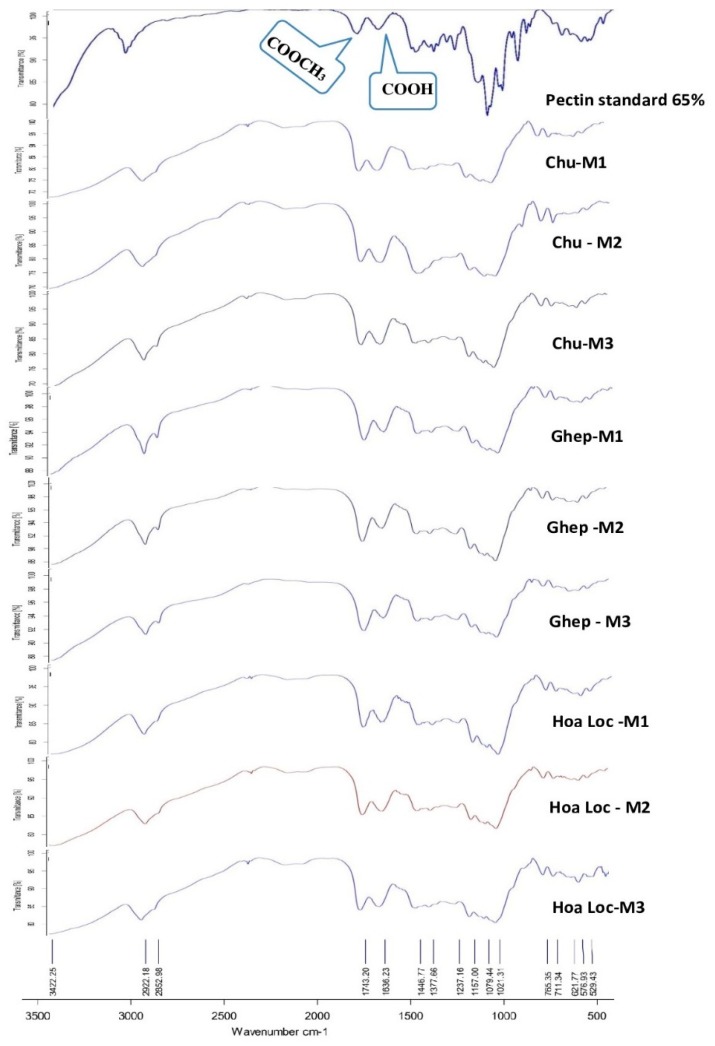
Fourier-transform infrared spectroscopy (FTIR) spectra of mango peel pectin collected from the three mango varieties at different maturity stages. Chu-M1: the immature Chu mango; Chu-M2: the mature Chu mango; Chu-M2: the overripe Chu mango; Ghep-M1: the immature Ghep mango; Ghep-M2: the mature Ghep mango; Ghep-M2: the overripe Ghep mango.

FTIR spectra analysis, shown in Figure 1, is not only used to determine degree of esterification but also to identify functional groups of the mango peel pectin collected. The functional groups of the pectin were identified based on their corresponding frequencies and the intensities of absorption [37]. Analysis of spectra showed that structural properties of the mango peel pectin samples were not significantly affected by the differences in cultivars and maturity stages (Figure 1). All FTIR spectra were similar and showing the broad and strong absorption areas between 3500 and 2500 cm^−1^, indicating for O−H stretching vibrations due to free and bound hydroxyl groups of carboxylic acid [37]. From 3000 cm^−1^ to 2800 cm^−1^, there were sharp bands representing C−H absorptions including CH, CH_2_, CH_3_ stretching. The presence of the ester carbonyl (C=O) groups and carboxylate stretching bands (COO–) were evidenced by stronger bands between 1760 and 1740 cm^−1^ and between 1640 and 1620 cm^−1^, respectively. It is obviously seen that for the stronger stretching peaks between 1760 and 1740 cm^−1^, higher DE values are obtained (Figure 1, Table 1).

### 3.6. Emulsifying Properties

Emulsifying properties include both emulsifying activity (EA) and emulsifying stability (ES). The emulsion activity of the pectin extracted from the three cultivars ranged from 11.8% to 34.2%. The emulsion activity did not increase as the cultivars matured but there were overall differences between the three cultivars (mean 32.0% for Ghep, 18.5% for Cat Chu and 30.8% for Hoa Loc). Lopez-Franco et al. [38] found that the presence of protein and polysaccharide moities in the pectin led to interactions between electrostatic and steric repulsion forces, forming an interfacial membrane around the oil droplets and this prevents their flocculation and coalesence. They are bound to the neutral sugar side chains of pectin and these act as the anchors that enable the pectin to form an emulsified system [39]. In this system, proteins are active on the oil-water interface and are responsible for the emulsifying activity of pectin [39]. As proteins are found in mango peels [40], the differences in emulsion properties of pectin between the mango cultivars might be due to differences in the protein content of the peels and the neutral sugar side chain in the pectin structure.

In the current work, the emulsion stability of the pectin extracted from the peels was very variable. It is interesting to note that overall the emulsion stability of the pectin extracted from the mature peels (mean 65.1%) was overall higher than for the other two maturity stages. The mean emulsion stability of the pectin extracted from the ripe peels was much lower (34.5%). This may be a consequence of the breakdown of protein [41] and the degradation of the neutral sugar side chain in the pectin molecules resulting from the activities of polygalacturonase and β-galactosidase [31] at this stage.

### 3.7. Rheological Properties

**Figure 2:**
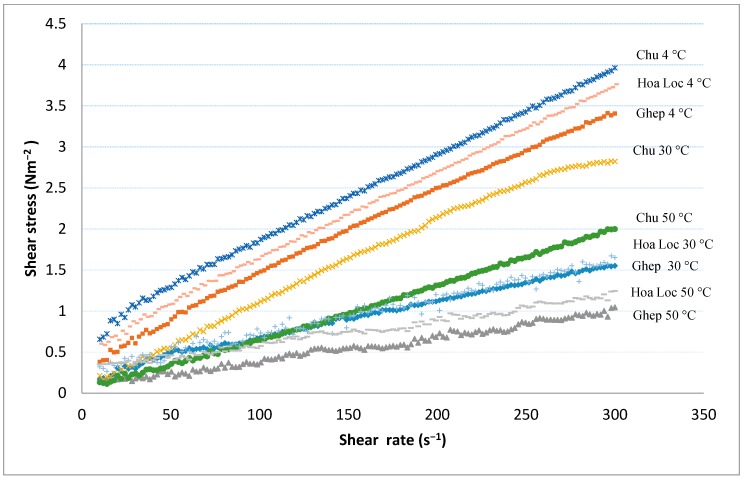
The effect of cultivars on viscosity of mango peel pectin solutions at different temperature.

The results showed that the viscosity of mango peel pectin was strongly dependent on the temperature (Figure 2). The highest viscosity was obtained from running at 4 °C, followed by 30 °C and 50 °C. At all studied temperatures, Chu pectin showed the highest viscosity, which seems to be affected by the molecular weight (Table 1).

### 3.8. Viscosity-Average Molecular Weight

In all studied varieties, the intrinsic viscosity and M_w_ fluctuated depending on the maturation (Table 1). They increased from pre-mature to mature and then reduced as fruit ripened. The highest values were from Cat Chu pectin at the mature stage. The phenomenon was in agreement with those obtained in banana and kiwi fruit [42,43]. The changes of the intrinsic viscosity and M_w_ could be due to pectinolytic enzymes including polygalacturonases and pectinmethylesterases [42]. As the M_w_ ranged from 397.0 to 578.0 kDa, the pectin of the three mango cultivars can be considered as medium weight molecules. It is interesting to note that pectin extracted from Ghep and Hoa Loc peels had the higher values of degree of esterification; however, they had lower intrinsic viscosity and average molecular weight as correspondingly compared to those of Cat Chu. Similar observations for apple pomace and citrus peel pectin were recorded by Owens et al. [44] and Morris et al. [45].

## 4. Conclusions

This is the first study to investigate the properties of crude pectin extracted from Vietnamese mango peels discarded as waste. It can be inferred from this study that the mango peel pectin isolated at different maturities and cultivars has a significant range of different characteristics and therefore the potential to have an important role in food processing. In particular, the overall mean high esterification value of the extracted pectin suggests that it should be classified as high methoxyl pectin, which is of considerable interest to food processors.

## Figures and Tables

**Table 1:** Properties of crude pectin extracted from peels of three Vietnamese mango cultivars at three maturity stages.

Cultivars Samples	Pectin Yield (g/100 g DW)	Degree of Esterification (%)	Water Holding Capacity (g H_2_O/1 g pectin)	Solubility (%)	Emulsion Activity (%)	Emulsion Stability (%)	Intrinsic Viscosity (mL/g)	M_w_ (kDa)
**Ghep**								
Pre mature	27.5 ± 1.2	55.8 ± 0.7	11.4 ± 0.2	81.7 ± 1.3	34.2 ± 0.3	65.8 ± 9.3	46.3 ± 0.1	397.0 ± 1.4
Mature	24.2 ± 0.7	55.8 ± 0.2	11.6 ± 0.8	87.4 ± 1.6	31.8 ± 1.7	40.2 ± 3.8	50.3 ± 0.2	444.5 ± 0.8
Ripe	31.7 ± 1.0	55.7 ± 0.5	13.6 ± 0.4	86.0 ± 1.4	29.9 ± 0.4	39.5 ± 3.4	50.1 ± 0.2	434.6 ±1.2
**Cat Chu**								
Pre mature	21.0 ± 0.7	50.9 ± 0.5	10.6 ± 0.3	77.4 ± 0.5	19.4 ± 4.0	35.5 ± 6.0	67.2 ± 0.3	539.8 ± 1.0
Mature	19.2 ± 0.4	49.6 ± 0.2	14.9 ± 1.1	79.1 ± 1.3	11.8 ± 0.9	60.6 ± 2.7	73.7 ± 0.4	578.0 ± 0.7
Ripe	26.5 ± 0.3	50.3 ± 0.5	9.5 ± 0.0	82.5 ± 1.2	24.2 ± 2.0	35.6 ± 0.6	71.3 ± 0.5	564.0 ± 1.5
**Hoa Loc**								
Pre mature	20.5 ± 0.9	52.1 ± 0.2	11.3 ± 0.3	83.4 ± 1.8	30.9 ± 1.1	85.5 ± 3.1	44.5 ± 0.2	408.2 ± 0.8
Mature	18.4 ± 0.8	52.4 ± 0.5	13.4 ± 0.6	85.1 ± 1.2	33.8 ± 0.5	94.5 ± 3.7	52.0 ± 0.3	444.6 ± 1.5
Ripe	24.1 ± 1.2	51.0 ± 0.3	11.8 ± 0.7	84.6 ± 1.3	27.6 ± 3.4	28.5 ± 5.0	50.3 ± 0.2	432.8 ± 1.1
Analysis of variance	Significance							
Cultivars	**	**	ns	**	**	*	*	*
Maturity stages	**	ns	*	**	ns	**	ns	ns
Interaction	**	ns	**	ns	**	**	*	*
LSD cultivars	3.1	0.8	---	2.8	4.6	21.2	1.7	9.9
LSD maturity	4.5	---	1.6	3.3	---	19.6	---	---

Significance: * *p* < 0.05; ** *p* < 0.01; Data are expressed as means ± standard error (*n* = 3). ns: not significant; LSD: Least Significant Difference. “---“: not calculated.
